# Ascl2 Knockdown Results in Tumor Growth Arrest by miRNA-302b-Related Inhibition of Colon Cancer Progenitor Cells

**DOI:** 10.1371/journal.pone.0032170

**Published:** 2012-02-23

**Authors:** Rong Zhu, Yongtao Yang, Yin Tian, Jianying Bai, Xin Zhang, Xiaohuan Li, Zhihong Peng, Yonghong He, Lei Chen, Qiong Pan, Dianchun Fang, Wensheng Chen, Chen Qian, Xiuwu Bian, Rongquan Wang

**Affiliations:** 1 Department of Gastroenterology, Southwest Hospital, Third Military Medical University, Chongqing, People's Republic of China; 2 Department of Pathology, Southwest Hospital, Third Military Medical University, Chongqing, People's Republic of China; University of Chicago, United States of America

## Abstract

**Background:**

Achaete scute-like 2 (Ascl2), a basic helix-loop-helix (bHLH) transcription factor, controls the fate of intestinal stem cells. However, the role of Ascl2 in colon cancer progenitor cells remains unknown. The cell line HT-29 (47.5–95% of CD133^+^ population) and LS174T (0.45% of CD133^+^ population) were chosen for functional evaluation of Ascl2 in colon cancer progenitor cells after gene knockdown by RNA interference.

**Methodology/Principal Findings:**

Immunohistochemistry demonstrated that Ascl2 was significantly increased in colorectal adenocarcinomas. Downregulation of Ascl2 using RNA interference in cultured colonic adenocarcinoma HT-29 and LS174T cells reduced cellular proliferation, colony-forming ability, invasion and migration in vitro, and resulted in the growth arrest of tumor xenografts in vivo. The Ascl2 protein level in CD133^+^ HT-29 cells was significantly higher than in CD133^−^ HT-29 cells. Ascl2 blockade via shRNA interference in HT-29 cells (shRNA-Ascl2/HT-29 cells) resulted in 26.2% of cells staining CD133^+^ compared with 54.7% in control shRNA-Ctr/HT-29 cells. The levels of ‘stemness’ associated genes, such as CD133, Sox2, Oct4, Lgr5, Bmi1, and C-myc, were significantly decreased in shRNA-Ascl2/HT-29 and shRNA-Ascl2/LS174T cells in vitro as well as in the corresponding tumor xenograft (CD133 was not performed in shRNA-Ascl2/LS174T cells). The shRNA-Ascl2/HT-29 cells had inhibited abilities to form tumorspheres compared with control. The microRNA (miRNAs) microarrays, identified 26 up-regulated miRNAs and 58 down-regulated miRNAs in shRNA-Ascl2/HT-29 cells. Expression levels of let-7b, miRNA-124, miRNA-125b, miRNA-17, miRNA-20a and miRNA-302b, involved in the regulation of ‘stemness’, were quantified with qPCR, which confirmed their identities. Restoration of miRNA-302b, via its mimic, led to the restoration of shRNA-Ascl2/HT-29 ‘stemness’ characteristics, including tumorsphere formation and ‘stemness’ associated genes levels, and the recovery of cellular behaviors, including colony-forming ability, invasion and migration in vitro.

**Conclusions/Significance:**

Ascl2 may be a potential target for the inhibition of colon cancer progenitor cells, and functions through a miR-302b-related mechanism.

## Introduction

Colorectal cancer (CRC), the third leading cause of death from cancer worldwide and a leading cause of morbidity and mortality in developed countries [1], represents a major therapeutic challenge for cancer. Recently, the cancer stem cell (CSC) hypothesis has been proposed to explain the functional heterogeneity and carcinogenesis of cancer. According to this model, a subpopulation of cancer cells, which exhibit stem-like features, sustain tumor formation, metastasis, and resistance to therapy [2]–[5]. In this respect, CSCs would be expected to have a stem cell-like/progenitor phenotype (generally referred to as “stemness”). Additionally, several studies have investigated the protein-coding genes and their products that participate in the stemness maintenance and tumorigenicity of colon cancer progenitor cells [6]–[8]. Thus, it is important to identify the regulatory mechanisms and signaling pathways involved in colon cancer progenitor cells to develop novel reagents to target the refractory colon cancer progenitor cells population [9].

Achaete scute-like 2 (Ascl2), a basic helix-loop-helix (bHLH) transcription factor, is a downstream target of Wnt signaling in intestinal stem cells. In situ hybridization demonstrated Ascl2 expression at the base of small and large intestinal crypts, but a lack of expression in other normal tissues, except placenta [10]. The combined results from these gain- and loss-of-function experiments imply that Ascl2 controls the fate of intestinal stem cells [11]. Several groups have demonstrated that Ascl2 is over-expressed in colorectal cancer [10], [12], [13]. Furthermore, Ascl2 over-expression has the potential to shift the hierarchy of stem and progenitor cells within liver metastases resulting in self-renewal rather than differentiation, potentially affecting the clinical behavior of these tumors [13]. Thus, Ascl2 may be a regulatory factor that controls the fate of colon cancer progenitor cells. During the past decade, a number of developmental pathways that regulate CSCs have been elucidated [14]–[17]. However, the role of Ascl2 in colon cancer progenitor cells remains unknown.

The cell line HT-29 has a CD133^+^ population of 47.5–95% in the literature [18], [19] and isolated CD133^+^ cells from the HT-29 colon cancer cell line exhibited a higher tumorigenic potential than CD133^−^ cells in the in vivo tumor formation assay [20]. The CD133 protein was first recognized as a surface marker for haematopoietic stem cells [21], later, it was used to recognize cancer stem cells in many solid tumors arising in, for example, breast [22], pancreas [23], liver [24] and colon [18], [19]. The cell line LS174T has a CD133^+^ population of 0.45% in the literature [20] and 0.1% in our experiment (data not shown). Thus HT-29 and LS174T cells were chosen for functional evaluation of Ascl2 in colon cancer progenitor cells after gene knockdown by RNA interference.

MicroRNAs (miRNAs) are crucial as post-transcriptional regulators of gene expression and participate in several biological functions, including cellular proliferation, differentiation and apoptosis [25]. miRNAs also contribute to preserving stemness of embryonic stem cells and human CSCs [26]–[28]. Investigation of the function of Ascl2 on colon cancer progenitor cells and miRNA expression profiles is crucial for elucidating the characteristics of colon cancer progenitor cells, which will benefit the development of new drugs or novel therapeutic methods that target colon cancer progenitor cells. Furthermore, it will provide new insights into methods to eradicate colon cancer due to the likelihood that the eradication of colon cancer progenitor cells will be a critical step in achieving a cure for colon cancer.

In this report, we demonstrate the selective blockade of Ascl2 in HT-29 and LS174T cells could inhibit cell growth, invasion and migration in vitro, and lead to growth arrest in vivo, that is partially related to miRNA-302b-related inhibition of ‘stemness’ of colon cancer progenitor cells based on the experiments of shRNA-Ascl2/HT-29 transfected with miRNA-302b mimic. These results indicate that Ascl2 could be a potential target in colon cancer progenitor cells for the development of novel therapies for the eradication of colon cancers.

## Materials and Methods

### Cell culture

Human colonic adenocarcinoma cell lines HT-29 and LS174T were obtained from the American Type Culture Collection (ATCC) and were maintained in McCoy's 5A medium (Sigma, USA) containing 10% fetal bovine serum (FBS; HyClone, USA) at 37°C and 5% CO_2_, with the medium changed every two days. Cells were passaged at 80% confluence and seeded at 30% confluence for maintenance of optimal proliferating conditions.

### RNA interference

The sequence, CCGCGTGAAGCTGGTGAAC, targeting Ascl2 (shRNA-Ascl2/EGFP) [11] was formed from duplex DNA consisting of 5′-CACCGCCGCGTGAAGCTGGTGAACTTCAAGACGGTTCACCAGCTTCACGCGGTTTTTTG-3′, 5′-AGCTCAAAAAACCGCGTGAAGCTGGTGAACCGTCTTGAAGTTCACCAGCTTCACGCGGC-3′.

A pGenesil-1.1 was used without insert for control (shRNA-Ctr/EGFP). The annealed DNA duplexes were cloned into the plasmid of pGenesil-1.1 digested with the Eco31I restriction enzyme. HT-29 and LS174T cells were transfected with shRNA-Ascl2/EGFP or shRNA-Ctr/EGFP vector, and then selected with 0.8 mg/ml G418 for HT-29 transfected cells and 0.4 mg/ml G418 for LS174T transfected cells, beginning 48 hours post transfection. Two weeks later, cells were maintained in 0.4 mg/ml G418 for HT-29 transfected cells and 0.2 mg/ml G418 for LS174T transfected cells until three independent stable transfected clones were established. RNA interference was stable throughout the span of experiments under the selection pressure of 0.4 mg/ml G418 for HT-29 transfected cells and 0.2 mg/ml G418 for LS174T transfected cells in the culture medium.

### Proliferation Assay

Cell proliferation was examined on days 1, 2, 3 and 4. Isolated cells were seeded at 1×10^4^ cells/well in 96-well plates (Corning, USA) in a final volume of 100 µl of culture medium per well. At each time point, 5 mg/ml MTT (Sigma, USA) was added to the culture medium (20 µl/well) and incubated for additional 4 hours at 37°C in 5% CO_2_ atmosphere to allow MTT to be converted to formazan crystals. After that, the formazan crystals were solubilized with 150 µl DMSO (Sigma, USA) for 10 min. The absorbance was measured at a wavelength of 490 nm with a microplate reader (Thermo, USA). All assays were repeated three times.

### Colony Formation Assay

Cells were plated at a density of 1000 cells per plate (35 mm, Corning, USA) and then incubated at 37°C under 5% CO_2_. The medium was changed every 3–4 days. On days 20, cells were stained with Giemsa and observed under an inverted microscope. The numbers of colonies in each plate were counted. The experiment was replicated three times and expressed as the average number of colonies per plate.

### In Vitro Invasion Assay

The in vitro invasion capability of the cells was measured using the transwell chambers coated with Matrigel (Corning, USA) assay. Cells were seeded in 100 µl at a density of 1×10^6^ cells/ml with 1% FBS in the upper chamber, and the lower chamber was filled with 600 µl of culture medium with 20% FBS as a chemoattractant. Transwells were then incubated at 37°C under 5% CO_2_ for 48 h to allow the cells to invade. At the end of the incubation, the cells on the upper side of the Matrigel-coated filter were removed by wiping with a cotton swab. Cells that had invaded through the Matrigel-coated filter were stained with crystal violet solution. The invasive cells that migrated through the Matrigel-coated filter to the lower surface were counted under an inverted light microscope (Olympus, Japan), at 200× magnification. Cells in five randomized fields of view at 200× were counted and expressed as the average number of cells per field of view. The experiment was replicated three times.

### Migration

HT-29, LS174T cells and their transfectants, at 90–100% confluence in 6-well plates, were cultured overnight in serum-free medium. The medium was replaced with PBS, and the monolayers were wounded mechanically using a sterilized, single-edged razorblade. After wounding, cells were rinsed twice with sterilized PBS and incubated in McCoy's 5A medium containing 10% FBS for 48 hours at 37°C, under 5% CO_2_. Cells that had migrated from the wounded edge were counted at 200× magnification using an inverted light microscope (Olympus, Japan). Cells in 5 random fields of view at 200× and expressed as the average number of cells per field of view. The experiment was replicated three times.

### Tumorsphere-formation assays

For tumorsphere formation, single-cell suspensions were suspended in a Dulbecco's Modified Eagle's Medium/F12 (DMEM/F12, Hyclone, USA) supplemented with B-27 (1×, Gibco), 20 ng/mL epidermal growth factor (EGF, Peprotech, USA), and 20 ng/mL basic fibroblast growth factor (bFGF, Peprotech, USA), and then plated in 24-well ultra-low attachment plates (Corning, USA) at a concentration of 1000 cells per well. Plates were analyzed 7–10 days later for tumorsphere formation and were quantified using an inverted microscope (Olympus) at 100× and 400× magnifications. For subsequent quantification of cell numbers per tumorsphere, tumorspheres were collected with a 40 um sieve (BD Biosciences, San Jose, CA, USA) and disassociated with 0.25% trypsin/0.02% EDTA to make a single cell suspension. The viable cells were then counted using trypan blue exclusion.

### In vivo tumorigenicity

The shRNA-Ctr/HT-29, shRNA-Ascl2/HT-29, shRNA-Ctr/LS174T and shRNA-Ascl2/LS174T cells were resuspended in 100 µl (1×10^6^ cells) of 0.9% physiological saline before injection. Six-week-old BALB/c nude male mice were purchased from the Animal Facility of Research Center of Third Military Medical University and maintained under standard conditions. All experiments were performed with the approval of the Animal Studies Ethics Committee of the Third Military Medical University (permit number: sw 20090713). Mice were subcutaneously inoculated with 1×10^6^ isolated cells on both flanks (the left with shRNA-Ascl2/HT-29 or shRNA-Ascl2/LS174T cells and the right with shRNA-Ctr/HT-29 or shRNA-Ctr/LS174T cells, respectively). The tumor sizes were measured using calipers. Tumor sizes were calculated using the formula: (length × width^2^)/2. The mice were euthanized by cervical dislocation on day 20 after inoculation. The grafts were removed, documented by photography and the tumor weights measured. Tumors were divided into two groups, and either fixed with 10% buffered formalin or preserved in −80°C.

### Flow cytometry cell sorting and flow cytometry analysis

For isolation of CD133^+^ and CD133^−^ populations within HT-29 cells, single-cell suspensions were incubated with phycoerythrin (PE)-conjugated anti-human CD133 antibody (AC133 clone; Miltenyi Biotec, Auburn, CA, USA) and FcR blocking reagent (Miltenyi Biotec, Auburn, CA, USA) in staining solution containing 0.5% BSA and 2 mM EDTA for 10 min at 4°C. Isotype-matched mouse immunoglobulin G1 (Miltenyi Biotec, Auburn, CA, USA) served as a negative control. Cells were analyzed and sorted with a fluorescence-activated cell sorter (FACS) (BD Biosciences, San Jose, CA, USA). For the positive and negative population, the top 10.8% brightly stained cells or the bottom 7.6% dimly stained cells were selected, respectively.

### Immunohistochemistry

Immunohistochemical studies of Ascl2 were performed on human colon mucosa (n = 11), colon carcinoma (n = 11) and tumor xenografts from nude mice (n = 6), the human colon mucosa and cancer tissues were from the same patients and all patients provided informed consent. Paraffin was removed from formalin-fixed, paraffin-embedded tissue; samples were then blocked and incubated with specific antibodies overnight at 4°C. The antibody was detected by SP9002 Histostain™-Plus Kits (Zymed Co., USA). All sections were counterstained with hematoxylin. Primary mouse Ascl2 monoclonal antibody (Table S1) was used at a dilution of 1∶100. All experiments were performed with the approval of the Studies Ethics Committee of Southwest Hospital, Third Military Medical University (permit number: sw 20090713).

### Immunofluorescence staining

HT-29 and LS174T cells, cultured on sterilized cover slips, were stained with Ascl2 primary antibody (Table S1), followed by incubation with goat anti-mouse IgG-R (Santa Cruz Biotechnology, Inc., California U.S.A.), counterstaining with DAPI, and finally visualized under a laser scanning confocal fluorescent microscope (Carl Zeiss, Inc. Germany).

### Real-time PCR analysis

Total RNA was extracted using TRIzol reagent (Invitrogen, USA) according to the manufacturer's instructions. First strand cDNA was synthesized using primeScript™ RT enzyme mix I, oligo dT primers and random hexamers (Takara, Japan). To determine fold changes in each gene, real-time PCR was performed using the first strand cDNA, forward and reverse primers, and the SYBR premix Ex Taq™ Green II (Takara, Japan). The primer sequences are summarized in Table S2. Reaction and signal detection were measured by the real-time PCR system (BioRad, USA). Expression levels were calculated as the relative expression ratio compared to β-actin. The real-time PCRs were performed in triplicate independently.

### Western blot assay

Cell lysates or the homogenized tissues from tumor xenografts dissolved in SDS sample buffer were separated by SDS-PAGE and transferred to nitrocellulose membrane. The β-actin was used as a control. The membrane was probed with specific primary antibody overnight at 4°C (the primary antibodies are summarized in Table S1), followed by incubation with HRP-conjugated secondary IgG(H+L) antibody (Santa Cruz Biotechnology, Inc., California U.S.A.). The blots were processed with Immobilon™ western chemiluminescent HRP substrate (Milipore, USA) and analyzed by gel imaging analysis system (BioRad, USA).

### miRNA microarray analysis and miRNAs quantification using quantitative PCR

The 6th generation of human miRNA microarrays (Exiqon, Denmark) was used to compare the miRNA expression profiles between shRNA-Ctr/HT-29 and shRNA-Ascl2/HT-29 cells. The microarray contains more than 1891 capture probes, covering all human, mouse and rat miRNAs annotated in the miRBase 16.0. Total RNA was extracted from 1×10^7^ stable transfected shRNA-Ctr/HT-29 and shRNA-Ascl2/HT-29 cells using TRIzol reagent (Invitrogen, USA). RNA isolation, quality control, labeling, and hybridization were performed at Shanghai KANCHENG Biochip Company according to the protocols in the miRNA microarray system. Arrays were scanned using a Microarray Scanner, and the scanned images were then imported into GenePix Pro 6.0 software (Axon) for grid alignment and data extraction. Replicated miRNAs were averaged and miRNAs with intensities >50 in all samples were chosen for calculating the normalization factor. Expressed data were normalized using the Median normalization. After normalization, differentially expressed miRNAs were identified through Fold Change filtering. Hierarchical clustering was performed using MEV software (v4.6, TIGR).

For miRNAs qPCR validation, the primer sequences for miRNAs are summarized in Table S3. RNA isolation, quality control, cDNA synthesize and qPCR were performed at Shanghai KANCHENG Biochip Company according to the protocols. A t-test was used to identify differentially expressed miRNAs between shRNA-Ctr/HT-29 and shRNA-Ascl2/HT-29 comparisons.

### Transfection of miRNA mimics or miRNA inhibitors in shRNA-Ascl2/HT-29 cells

shRNA-Ascl2/HT-29 cells were transfected 24 hours after being seeded in 6-well plates. The miRNA mimics (100 pmol) or miRNA inhibitors (200 pmol) (Guangzhou Ribobio Co., Ltd. Guangzhou, PR of China) in 250 µl of serum-free, antibiotics-free medium were mixed with 5 µl of Lipofectamine 2000 transfection reagent (Invitrogen, Carlsbad, CA) dissolved in 245 µl of the same medium and allowed to stand at room temperature for 20 min. The resulting 500 µl transfection solutions were then added to each well containing 1.5 ml of medium. Six hours later, each well was replaced with 2 ml fresh medium supplemented with 10% FBS. The transient transfected cells were collected after an additional 48 hours of incubation for further experiments, including the tumorsphere formation, real-time PCR, western blot analysis, colony formation assay, invasion assay and migration analysis. The transient transfected shRNA-Ascl2/HT-29 cells using miRNA mimic negative control (100 pmol) or miRNA inhibitors negative control (200 pmol) were used as a control.

### Statistical Analysis

For continuous variables, data were expressed as the mean ± standard deviation. Differences between groups were estimated by Student's t-test and repeated-measures ANOVA analysis. All differences were deemed significant at the level of p<0.05, very significant at the level of p<0.01. Statistical analyses were performed by the SPSS 13.0 for Windows software package.

## Results

### Ascl2 is overexpressed in colon cancer and colon cancer cell lines, and Ascl2 interference in HT-29 and LS174T cells remarkably reduced its expression

**Figure 1:**
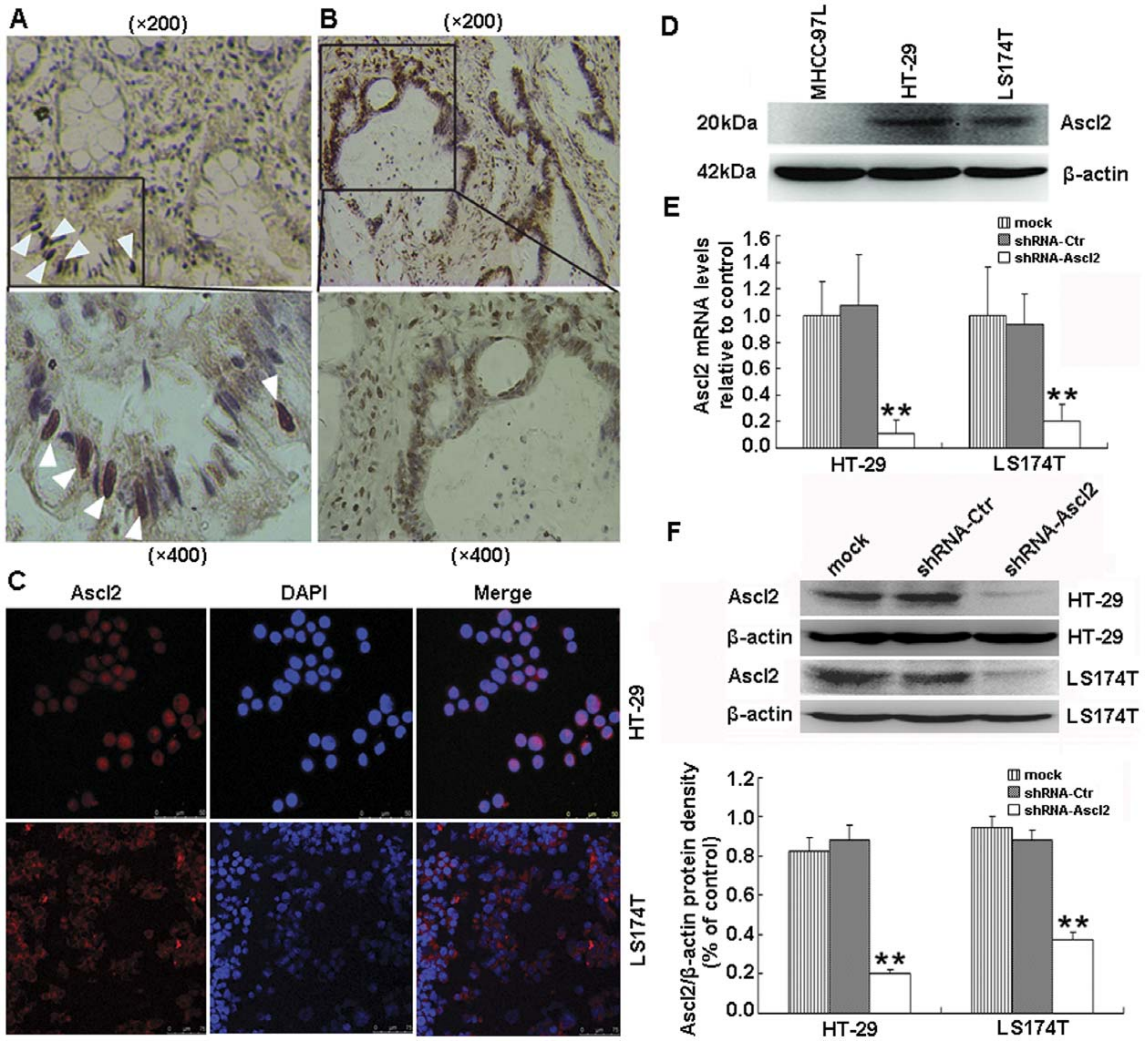
Expression of Ascl2 in normal human colon mucosa, colon cancer and colon cancer cell lines and its selective interference in HT-29 and LS174T cells. Ascl2 expression is specifically localized at the nucleus of crypt base cells of normal human colon mucosa (arrowhead) (A), B demonstrates that Ascl2 is specifically expressed at the nucleus of human colon cancer cells in human colon cancer tissues (Original magnification of top panel of A and B: ×200; Original magnification of low panel of A and B: ×400). The immunofluorescent staining indicates Ascl2 located mainly in the nucleus of HT-29 cells and the cytoplasm of LS174T cells (C) (Original magnification: ×200). Western blot analysis shows Ascl2 is present in HT-29 and LS174T cells, but absent in MHCC-97L cells (D). Ascl2 interference in HT-29 and LS174T cells results in the significant reduction of both Ascl2 mRNA analyzed by real-time PCR and protein levels analyzed by western blot analysis relative to control (β-actin) (**: p<0.01) (E and F).

Immunohistochemical staining was used to determine whether Ascl2 protein was expressed in human colon mucosa and colon cancer. An increase in Ascl2 protein expression in the nucleus of colon cancer cells of human colon cancers (Figure 1B) was observed when compared with the specific staining of Ascl2 protein at the nucleus of crypt base cells of normal colon mucosa (Figure 1A). The cells with Ascl2 positive staining in the nucleus were separated by negative staining cell exactly in the normal crypt base (Figure 1A). The immunofluorescence staining demonstrated that Ascl2 was expressed mainly in the nucleus of HT-29 cells, and weakly expressed in the cytoplasm, whereas, Ascl2 was expressed mainly in the cytoplasm of LS174T cells, and weakly expressed in the nucleus. Its expression pattern of Ascl2 in HT-29 and LS174T cells was discordant, with the majority of HT-29 and LS174T cells being relatively weak for expression (Figure 1C). Western blot analysis demonstrated that Ascl2 was present in both colon cancer cell lines HT-29 and LS174T cells (20 kDa in both), but absent in MHCC-97L, a liver cancer cell line (Figure 1D).

To knock down Ascl2 expression in HT-29 and LS174T cells, cells were transfected with shRNA-Ascl2/EGFP and shRNA-Ctr/EGFP vectors, four stable-transfected cell lines (shRNA-Ascl2/HT-29, shRNA-Ascl2/LS174T, shRNA-Ctr/HT-29 and shRNA-Ctr/LS174T cells) were established. Ascl2 mRNA quantitated with real-time PCR was reduced in shRNA-Ascl2/HT-29 and shRNA-Ascl2/LS174T cells compared with their controls (Figure 1E). A corresponding reduction in Ascl2 protein was observed in shRNA-Ascl2/HT-29 and shRNA-Ascl2/LS174T cells compared with their controls (Figure 1F). There was no significant difference between shRNA-Ctr/HT-29 and untransfected HT-29 cells, and between shRNA-Ctr/LS174T and untransfected LS174T cells, in both Ascl2 mRNA and protein expression (Figure 1E and 1F).

### Silence of Ascl2 in HT-29 and LS174T cells led to alterations in cellular behaviors

**Figure 2:**
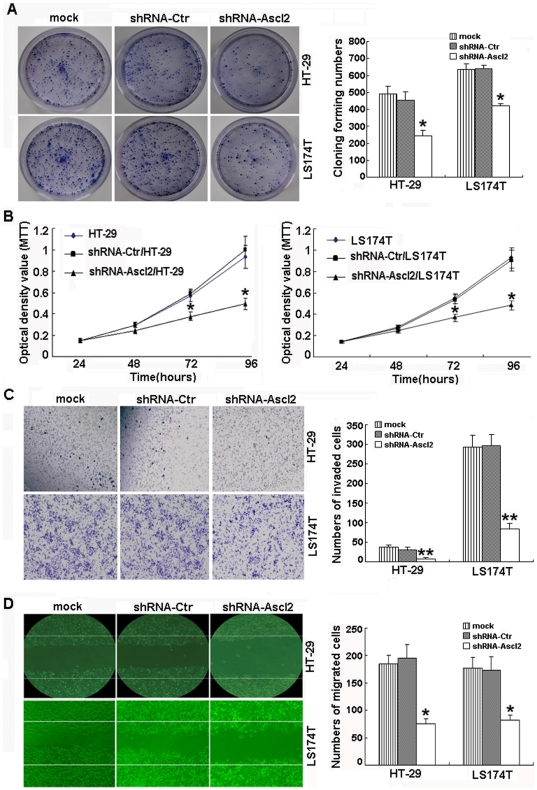
Ascl2 blockade in HT-29 and LS174T cells results in the inhibition of colony formation, proliferation, invasion and migration in vitro. shRNA-Ascl2/HT-29 and shRNA-Ascl2/LS174T cells have fewer colonies (*: p<0.05) (A), lower growth rates (*: p<0.05) (B), less invaded cells through the Matrigel-coated membrane (**: p<0.01) (C) and less cells migrating across the scraped edge (*: p<0.05) (D), when compared with their controls (Original magnification: ×200).

Colony formation assay: shRNA-Ascl2/HT-29 and shRNA-Ascl2/LS174T cells developed fewer colonies after 20 days compared with their controls (p<0.05) (Figure 2A). Proliferation assay: The proliferation rates of shRNA-Ascl2/HT-29, shRNA-Ascl2/LS174T and their controls were examined using an MTT method, according to the manufacturer's protocol, from days 1 to 4 after seeding. As shown in Figure 2B, there were significant differences in the growth rates between shRNA-Ascl2/HT-29 and HT-29 cells, and between shRNA-Ascl2/HT-29 and shRNA-Ctr/HT-29 cells at days 3 and 4, also between shRNA-Ascl2/LS174T and LS174T cells, and between shRNA-Ascl2/LS174T and shRNA-Ctr/LS174T cells at days 3 and 4 (p<0.05). In vitro invasion assay: Invasion assays were performed using Matrigel-coated transwell culture chambers. After 48 hours of incubation, the numbers of invading cells were counted. With shRNA-Ascl2/HT-29 cells, 7±3 cells per field (under 200×magnification using the inverted microscope) invaded through the membrane. This number was significantly lower than that of untransfected HT-29 cells (37±5) or shRNA-Ctr/HT-29 cells (31±6) (p<0.01). Similarly, with shRNA-Ascl2/LS174T cells, 84±14 cells per field (under 200×magnification using the inverted microscope) invaded through the membrane. This number was significantly lower than that of untransfected LS174T cells (292±32) or shRNA-Ctr/LS174T cells (296±30) (p<0.01) (Figure 2C). Migration: Finally, 75±10 shRNA-Ascl2/HT-29 cells per field (under 200× magnification) moved across the scraped edge after 48 hours. This number was significantly lower than that of untransfected HT-29 cells (185±15) or shRNA-Ctr/HT-29 cells (195±25) (p<0.05). 82±9 shRNA-Ascl2/LS174T cells per field (under 200× magnification) moved across the scraped edge after 48 hours. This number was significantly lower than that of untransfected LS174T cells (177±21) or shRNA-Ctr/LS174T cells (173±25) (p<0.05) (Figure 2D).

### Ascl2 interference led to in vivo growth arrest of tumors

**Figure 3:**
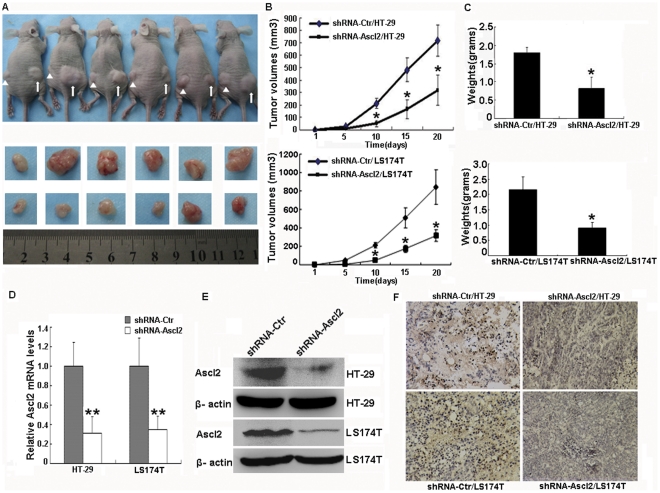
Ascl2 interference in HT-29 and LS174T cells leads to tumor growth arrest in vivo. All mice (6/6, respectively) develop tumors 20 days later after 1×10^6^ shRNA-Ctr/HT-29 cells (right side and marked as arrow) and shRNA-Ascl2/HT-29 cells (left side and marked as arrowhead) are inoculated into nude mice (A), LS174T cells were not shown. The tumor volume (B) and mass weight (C) in the group of shRNA-Ascl2/HT-29 and shRNA-Ascl2/LS174T cells is significantly lower than the group of shRNA-Ctr/HT-29 and shRNA-Ctr/LS174T cells (*: p<0.05). The mRNA (D) and protein (E) levels of Ascl2 in the tumor tissues develop from shRNA-Ascl2/HT-29 and shRNA-Ascl2/LS174T cells are lower than in the tumor tissues developed from shRNA-Ctr/HT-29 and shRNA-Ctr/LS174T cells (**: p<0.01). Ascl2 immunostaining in the nucleus of the cancerous cells in tumor xenografts from shRNA-Ctr/HT-29 and shRNA-Ctr/LS174T cells is stronger than in the nucleus of the cancerous cells in tumor xenografts from shRNA-Ascl2/HT-29 and shRNA-Ascl2/LS174T cells (F).

To compare the growth rates of shRNA-Ctr/HT-29 and shRNA-Ascl2/HT-29 cells, shRNA-Ctr/LS174T and shRNA-Ascl2/LS174T cells, in athymic nude mice, the shRNA-Ascl2/HT-29 or shRNA-Ascl2/LS174T cells were injected into left flank while shRNA-Ctr/HT-29 or shRNA-Ctr/LS174T cells were injected into the right flank respectively. Twenty days after inoculation with each paired cells (shRNA-Ascl2/HT-29 and shRNA-Ctr/HT-29 cells in Figure 3A, or shRNA-Ascl2/LS174T and shRNA-Ctr/LS174T cells (results not shown)) in each mouse, respectively, all mice (12/12, respectively) developed tumors. Tumor volumes were measured using calipers at various time points before death, while weights were determined after death. The volume and mass in the shRNA-Ascl2/HT-29 or shRNA-Ascl2/LS174T tumors were significantly lower than that of the shRNA-Ctr/HT-29 or shRNA-Ctr/LS174T tumors (p<0.05) (Figure 3B and 3C). Furthermore, Ascl2 expression in the tumor tissue from shRNA-Ascl2/HT-29 or shRNA-Ascl2/LS174T cells was significantly lower than that of shRNA-Ctr/HT-29 or shRNA-Ctr/LS174T cells, as determined by real-time PCR and western blot analysis (Figure 3D and 3E). Ascl2 expression in tumor tissue from shRNA-Ascl2/HT-29 or shRNA-Ascl2/LS174T cells was significantly lower than that from shRNA-Ctr/HT-29 or shRNA-Ctr/LS174T cells, as shown by immunohistochemical staining (Figure 3F).

### Ascl2 expression in CD133^+^ and CD133^−^ HT-29 cells

**Figure 4:**
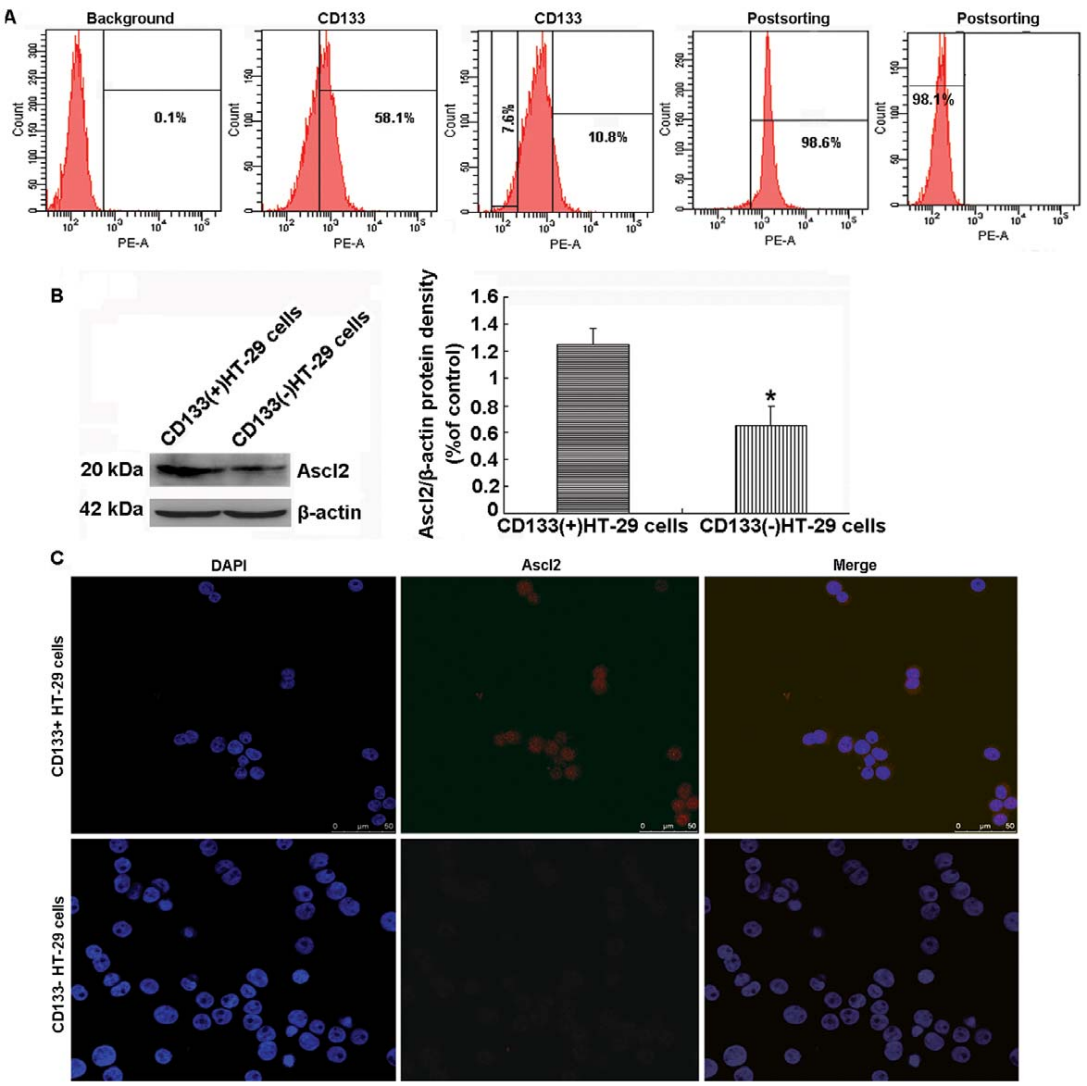
Sorting of HT-29 cells based on CD133 marker and Ascl2 expression in CD133^+^ and CD133^−^ HT-29 cells. CD133 is expressed in 58.1% of HT-29 cells. 98.6% of cells are confirmed to be CD133 positive by post-sorting selection in the top 10.8% of CD133^+^ HT-29 cells, 98.1% of cells are confirmed to be CD133 negative by post-sorting selection in the dimly 7.6% of CD133^−^ HT-29 cells (A). Ascl2 protein level relative to control (β-actin) in CD133^+^ HT-29 cells is significantly higher than that in CD133^−^ HT-29 cells (*: p<0.05) (B). There is an obvious nuclear staining of Ascl2 in CD133^+^ HT-29 cells, but Ascl2 is almost negative in CD133^−^ HT-29 cells (C) (Original magnification: ×200).

Flow cytometry analysis indicated CD133 was expressed in 58.1% of the HT-29 cells, with 0.1% HT-29 cells were detected in the negative control. 98.6% of cells were confirmed to be CD133 positive in the top 10.8% of CD133 positive HT-29 cells by postsorting selection, 98.1% of cells were confirmed to be CD133 negative in the dimly 7.6% of CD133 negative HT-29 cells (Figure 4A).

The CD133^+^ and CD133^−^ HT-29 cells were analyzed for the expression of Ascl2 using western blot assays. The Ascl2 protein level relative to control (β-actin) in CD133^+^ HT-29 cells was significantly higher than that in CD133^−^ HT-29 cells (p<0.05) (Figure 4B). The CD133^+^ and CD133^−^ HT-29 cells were immunostained with anti-Ascl2 antibody, demonstrating a nuclear staining pattern of Ascl2 in CD133^+^ HT-29 cells (the top panel of Figure 4C), and a negligible staining for Ascl2 in CD133^−^ HT-29 cells (the low panel of Figure 4C).

### The percentage of CD133^+^ HT-29 cells and ‘stemness’ markers were remarkably reduced following Ascl2 knockdown

**Figure 5:**
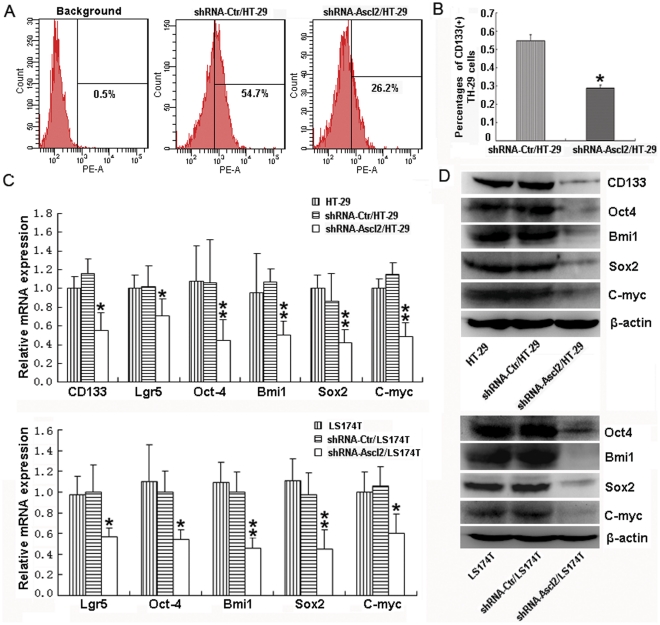
The percentage of CD133^+^ HT-29 cells and the expression level of ‘stemness’ associated genes are reduced due to Ascl2 knockdown in vitro. 54.7% of shRNA-Ctr/HT-29 cells are positive for CD133 expression compared with 26.2% of HT-29 cells are positive for CD133 expression in shRNA-Ascl2/HT-29 cells (*: p<0.05) (A and B). The mRNA levels of ‘stemness’ associated genes, like CD133 (not performed in LS174T cells and its transfectants), Lgr5, Oct4, Bmi1, Sox2, and C-myc analyzed by real-time PCR in the shRNA-Ascl2/HT-29 and shRNA-Ascl2/LS174T cells are lower than in the shRNA-Ctr/HT-29, HT-29, shRNA-Ctr/LS174T and LS174T cells, respectively (*: p<0.05; **: p<0.01) (C). The protein levels of ‘stemness’ associated genes, like CD133 (not performed in LS174T cells and its transfectants), Oct4, Bmi1, Sox2, and C-myc, analyzed by western blot analysis in shRNA-Ascl2/HT-29 and shRNA-Ascl2/LS174T cells are significantly lower than that in the shRNA-Ctr/HT-29, HT-29, shRNA-Ctr/LS174T and LS174T cells, respectively (D).

The observation that Ascl2 expression was higher in CD133^+^ HT-29 cells than in CD133^−^ HT-29 cells, led to the hypothesis that the Ascl2 protein is important for the expression of CD133 in CD133^+^ stem/progenitor HT-29 cells. The comparative flow cytometry analysis of shRNA-Ctr/HT-29 and shRNA-Ascl2/HT-29 cells was performed. In the shRNA-Ctr/HT-29 cells, 54.7% of the cells were positive for CD133 expression, whereas 26.2% of the cells were positive for CD133 expression in shRNA-Ascl2/HT-29 cells (Figure 5A). Triple experiments, using three different clones, confirmed that the percentage of CD133^+^ HT-29 cells was significantly reduced in the presence of Ascl2 knockdown (Figure 5B) (p<0.05). Only 0.1% of LS174T cells were CD133 positive in our own data and 0.45% of LS174T cells were CD133 positive in literarure [20] with flow cytometry sorting, so the comparative flow cytometry analysis of shRNA-Ctr/LS174T and shRNA-Ascl2/LS174T cells was not performed.

**Figure 6:**
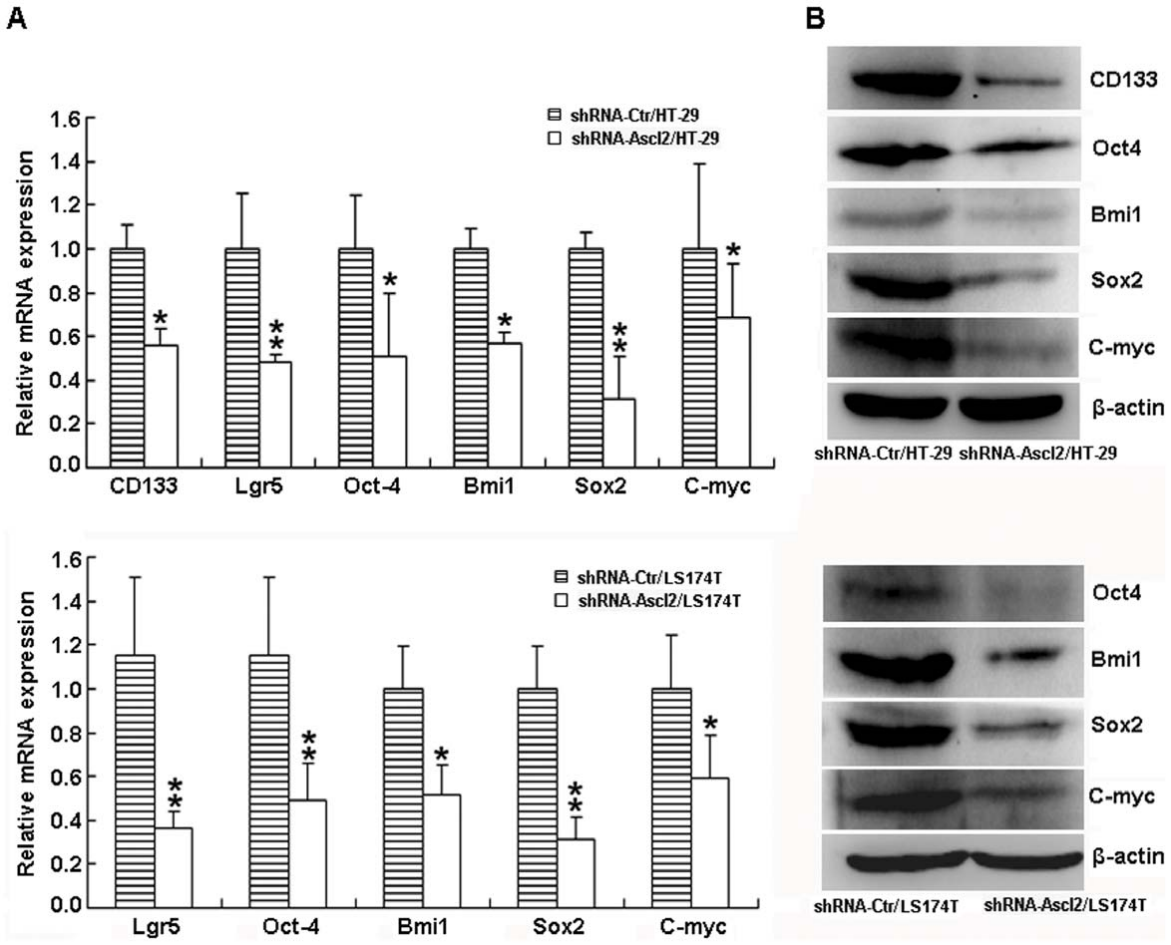
The expression level of ‘stemness’ associated genes are reduced due to Ascl2 knockdown in vivo. The mRNA levels of CD133 (not performed in the tumor tissues developed from shRNA-Ascl2/LS174T and shRNA-Ctr/LS174T), Lgr5, Oct4, Bmi1, Sox2, and C-myc, analyzed by real-time PCR in the tumor tissues developed from shRNA-Ascl2/HT-29 and shRNA-Ascl2/LS174T cells are lower than in the tumor tissues developed from shRNA-Ctr/HT-29 and shRNA-Ctr/LS174T cells (*: p<0.05; **: p<0.01) (A). The protein levels of CD133 (not performed in the tumor tissues developed from shRNA-Ascl2/LS174T and shRNA-Ctr/LS174T), Oct4, Bmi1, Sox2, and C-myc, analyzed by western blot analysis in the tumor tissues developed from shRNA-Ascl2/HT-29 and shRNA-Ascl2/LS174T cells are significantly lower than in the tumor tissues developed from shRNA-Ctr/HT-29 and shRNA-Ctr/LS174T cells (B).

To confirm whether Ascl2 interference in HT-29 or LS174T cells inhibited the “stemness” of progenitor cells present in the repertoire of HT-29 or LS174T cells, the expression of present candidate genes involved in stem cells was investigated by real-time PCR in shRNA-Ctr/HT-29 and shRNA-Ascl2/HT-29 cells, and in shRNA-Ctr/LS174T and shRNA-Ascl2/LS174T cells. There was a significant decrease in expression of mRNA levels of ‘stemness’ genes, such as CD133 (not preformed in shRNA-Ctr/LS174T and shRNA-Ascl2/LS174T cells), Lgr5, Oct4, Bmi1, Sox2, and C-myc in shRNA-Ascl2/HT-29 and shRNA-Ascl2/LS174T cells compared with shRNA-Ctr/HT-29 and shRNA-Ctr/LS174T cells (Figure 5C). The protein levels of ‘stemness’ genes, including CD133 (not preformed in shRNA-Ctr/LS174T and shRNA-Ascl2/LS174T cells), Oct4, Bmi1, Sox2 and C-myc, analyzed by western blot analysis (Western blot of Lgr5 protein was not successful when using the commercial antibodies), were significantly lower in shRNA-Ascl2/HT-29 and shRNA-Ascl2/LS174T cells compared with shRNA-Ctr/HT-29 and shRNA-Ctr/LS174T cells (Figure 5D). However, there was no significant difference in the expression of ‘stemness’ associated genes between shRNA-Ctr/HT-29 and non-transfected HT-29 cells, and between shRNA-Ctr/LS174T and non-transfected LS174T cells (Figure 5C and 5D). Similar experiments were performed in the tumor xenografts developed from shRNA-Ctr/HT-29, shRNA-Ascl2/HT-29 cells, shRNA-Ctr/LS174T or shRNA-Ascl2/LS174T cells. As shown in Figure 6A and 6B, the mRNA and/or protein levels of ‘stemness’ associated genes, including CD133 (not preformed in shRNA-Ctr/LS174T and shRNA-Ascl2/LS174T cells), Bmi1, Oct4, Sox2, Lgr5 and C-myc were significantly reduced in tumor xenografts developed from shRNA-Ascl2/HT-29 or shRNA-Ascl2/LS174T cells compared with tumor xenografts developed from shRNA-Ctr/HT-29 or shRNA-Ctr/LS174T cells.

### Knockdown of Ascl2 negatively regulated tumorsphere formation

**Figure 7:**
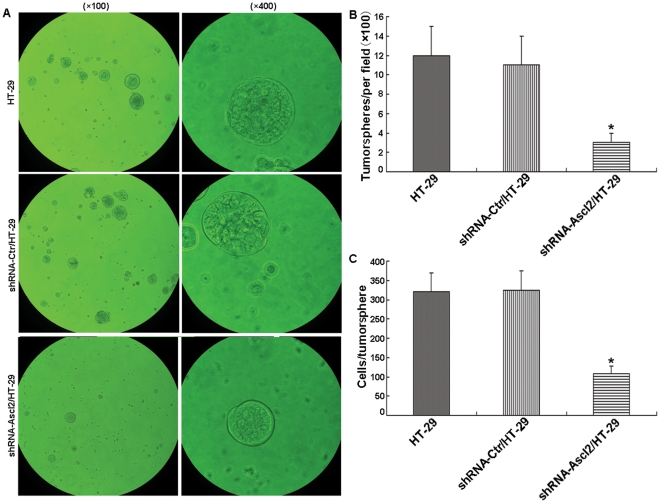
Knockdown of Ascl2 leads to the inhibition of the tumorsphere formation. 1000 cells of shRNA-Ascl2/HT-29, shRNA-Ctr/HT-29 and non-transfected HT-29 cells are plated for tumorsphere formation as described in Materials and methods and quantified at 100× and 400× magnifications (A). The number of tumorspheres from shRNA-Ascl2/HT-29 cells is significantly less than that from shRNA-Ctr/HT-29 and non-transfected HT-29 cells (*: p<0.05) (B). The number of cells per tumorsphere from shRNA-Ascl2/HT-29 cells is significantly less than that from shRNA-Ctr/HT-29 cells and non-transfected HT-29 cells (*: p<0.05) (C).

To examine the self-renewal potential of HT-29 cells with or without Ascl2 knockdown, we undertook tumorsphere formation culture of shRNA-Ascl2/HT-29, shRNA-Ctr/HT-29 and untransfected HT-29 cells in a special ultra-low attachment culture plate with conditional medium for tumorsphere formation (LS174T cells and its transfectants were not used for further experiments due to the failure of tumorsphere formation when using LS174T cells). HT-29 cells were not sorted for CD133 positivity prior to plating, for the following two reasons: first, more than 54.7% of the HT-29 cells are CD133^+^, second, Ascl2 knockdown in HT-29 cells led to a significant reduction in the percentage of CD133^+^ HT-29 cells. Thus, 7–10 days later, plates were analyzed for tumorsphere formation and were quantified using an inverted microscope. As shown in Figure 7, the significantly fewer and smaller tumorspheres were observed from shRNA-Ascl2/HT-29 cells than those from HT-29 cells and shRNA-Ctr/HT-29 cells (p<0.05, Figure 7A and 7B). The number of cells per tumorsphere from shRNA-Ascl2/HT-29 cells was significantly less than that from shRNA-Ctr/HT-29 cells and non-transfected HT-29 cells (p<0.05, Figure 7C).

### Transfection of miR-302b mimic in shRNA-Ascl2/HT-29 cells led to the recovery of the tumorsphere formation and expression of ‘stemness’ markers

**Figure 8:**
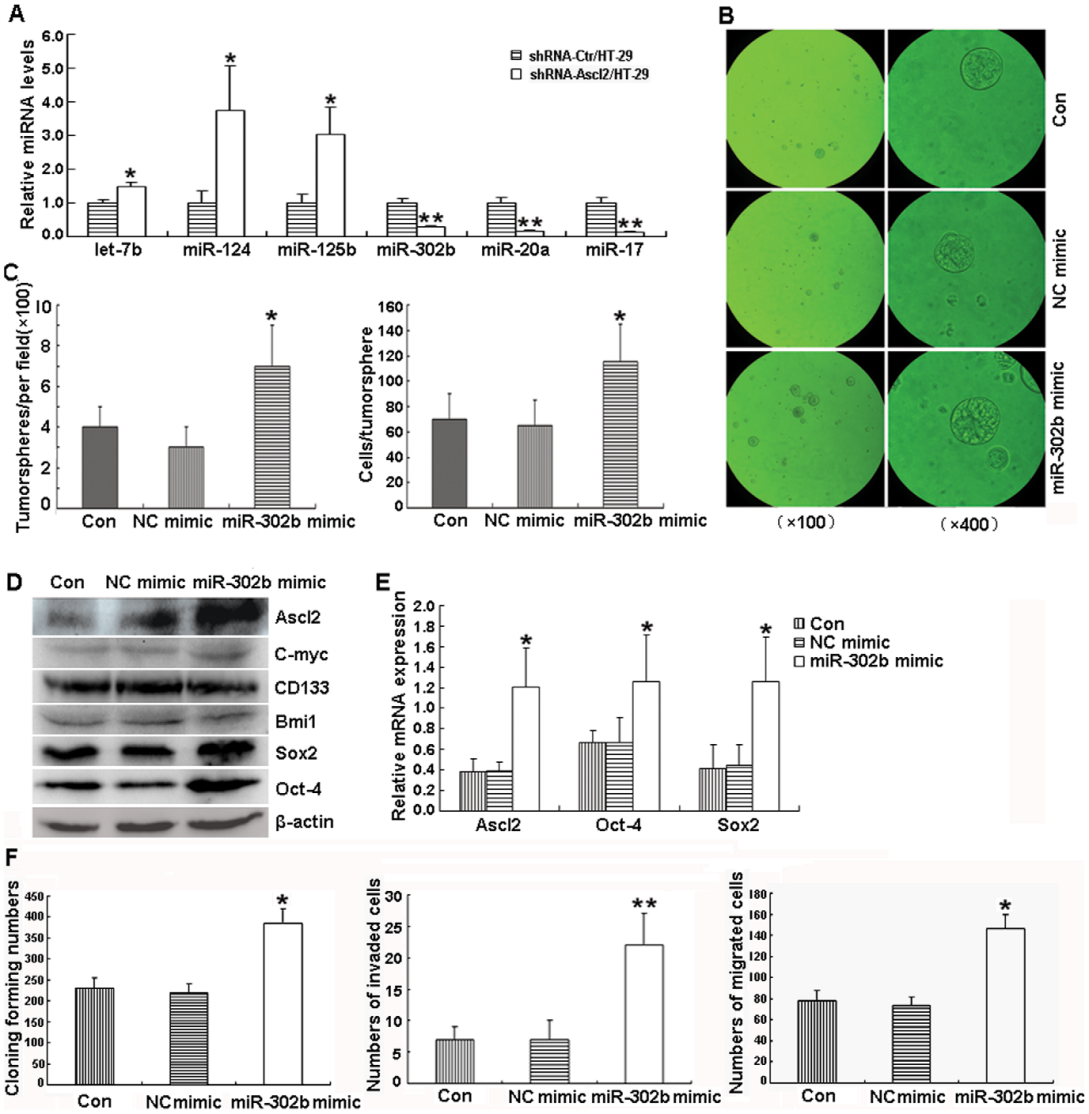
Transfection of miR-302b mimic in shRNA-Ascl2/HT-29 cells restores their ‘stemness’ characteristics and recovers their cellular behaviors in vitro. The let-7b, miRNA-124 miRNA-125b are significantly up-regulated, the miRNA-302b, miRNA-20a and miRNA-17 are significantly down-regulated, in shRNA-Ascl2/HT-29 cells compared with shRNA-Ctr/HT-29 cells (*: p<0.05;**: p<0.01) (A). The native shRNA-Ascl2/HT-29 cells, shRNA-Ascl2/HT-29 cells transfected with NC mimic and shRNA-Ascl2/HT-29 cells transfected with miR-302b mimic were analyzed for tumorsphere formation and were quantified at 100× (left panel of B) and 400× (right panel of B) magnifications. The number of tumorspheres and cells per tumorsphere from shRNA-Ascl2/HT-29 cells transfected with miR-302b mimic were significantly higher than those from shRNA-Ascl2/HT-29 and shRNA-Ascl2/HT-29 cells transfected with NC mimic (*: p<0.05) (C). Western blot analysis of Ascl2, C-myc, CD133, Bmi1, Sox2 and Oct4 in the cell lysates indicate that Ascl2, Sox2 and Oct4 protein levels are induced due to miR-302b mimic transfection in shRNA-Ascl2/HT-29 cells compared with shRNA-Ascl2/HT-29 and shRNA-Ascl2/HT-29 cells transfected with NC mimic (D). The real time PCR experiments for quantification of Ascl2, Oct4 and Sox2 mRNAs demonstrate a significant increase due to miR-302b mimic transfection of shRNA-Ascl2/HT-29 cells (*: p<0.05) (E). The colony-forming ability, the numbers of invaded cells and migrated cells of shRNA-Ascl2/HT-29 cells transfected with miR-302b mimic were significantly increased comparing with control (*: p<0.05) (F).

To determine the role of miRNAs during Ascl2-mediated regulation of CD133^+^ HT-29 cells and their ‘stemness’, the shRNA-Ctr/HT-29 and shRNA-Ascl2/HT-29 cells were analyzed using miRNA microarray studies. There were 84 differentially expressed miRNAs comprising 26 miRNAs that were 2.0 fold up-regulated and 58 miRNAs that were 2.0 fold down-regulated in shRNA-Ascl2/HT-29 cells compared with shRNA-Ctr/HT-29 cells (Tables S4 and S5). The data of the microarray analysis has been deposited in GEO DataSets (The GEO accession number: GSE34926). Notably, Expression levels of the up-regulated let-7b, miRNA-124 and miRNA-125b, and the downregulated miRNA-17, miRNA-20a and miRNA-302b, involved in the regulation of ‘stemness’, were quantified with qPCR, which confirmed their identities (Figure 8A).

To explore the mechanism of let-7b, miRNA-124, miRNA-125b, miRNA-17, miRNA-20a and miRNA-302b in the regulation of tumorsphere formation of the shRNA-Ascl2/HT-29 cells, miRNA inhibitors or mimics were transfected into shRNA-Ascl2/HT-29 cells. No significant difference in tumorsphere formation was observed after transfection with the let-7b, miR-124 and miR-125b inhibitors as well as the miR-17 and miR-20a mimics in shRNA-Ascl2/HT-29 cells (data not shown). However, the transfection of the miR-302b mimic into shRNA-Ascl2/HT-29 cells produced results.

The tumorspheres from shRNA-Ascl2/HT-29 cells and shRNA-Ascl2/HT-29 cells transfected with negative control mimic (NC mimic) were significantly fewer and smaller than shRNA-Ascl2/HT-29 cells transfected with miR-302b mimic (Figure 8B). The number of tumorspheres and cells per tumorsphere from shRNA-Ascl2/HT-29 cells transfected with miR-302b mimic were significantly higher than those from shRNA-Ascl2/HT-29 and shRNA-Ascl2/HT-29 cells transfected with NC mimic (Figure 8C). Ascl2, Oct4 and Sox2 protein and mRNA levels were induced following miR-302b mimic transfection in shRNA-Ascl2/HT-29 cells compared with shRNA-Ascl2/HT-29 and shRNA-Ascl2/HT-29 cells transfected with NC mimic (Figure 8D and 8E). The results indicate that miR-302b is an important miRNA related to the inhibition of cancer progenitor cells caused by Ascl2 selective blockade in HT-29 cells, which led to tumor growth arrest in vivo and in vitro.

### Transfection of miR-302b mimic in shRNA-Ascl2/HT-29 cells led to the increase of colony-forming ability, invasion and migration in vitro

As shown in Figure 8F, the colony-forming numbers, invaded cells through the Matrigel-coated filter and migrated cells from the wounded edge of shRNA-Ascl2/HT-29 cells transfected with miR-302b mimic were significantly more than shRNA-Ascl2/HT-29 cells transfected with NC mimic and non-transfected shRNA-Ascl2/HT-29 cells. It is possible that miR-302b is an important miRNA related to the inhibition of cellular behaviors of shRNA-Ascl2/HT-29 cells in vitro. Thus, we concluded that Ascl2 knockdown results in tumor growth arrest by miRNA-302b-related inhibition of colon cancer progenitor cells.

## Discussion

CD133 is regarded as a currently acceptable marker for the isolation and identification of the CSCs in both primary colon cancer and colon cancer cell lines [4], [29]. CD133 was chosen as marker of CSCs primarily due to its robust, heterogeneous expression by FACS, which enables the sorting of CD133^+^ and CD133^−^ populations, providing an ability for comparison with the additional CSC identification cell surface markers [8]. Due to the limitations of high heterogeneity and variability of primary CSCs isolated from patients, human cancer cell lines provide a new and stable genetic tumor material for the study of the fundamental features of CSCs. Therefore, the human colonic adenocarcinoma cell line HT-29 (47.5–95% of CD133^+^ population) was chosen as a model for this study and comparing with human colonic adenocarcinoma cell line LS174T (0.45% of CD133^+^ population).

CSCs are a subpopulation of tumor cells that possess the stem cell properties of self-renewal and differentiation. Stem cells may be the target cells responsible for malignant transformation, and tumor formation may be a disorder of the stem cell self-renewal pathway. The CSC theory clarifies the issues of tumor initiation, development, metastasis and relapse, as well as the ineffectiveness of conventional cancer therapies. Treatments directed against the bulk of cancer cells may produce striking responses but are unlikely to result in long-term remissions if the rare CSCs are not targeted. Therefore, targeting CSCs is an attractive and novel therapeutic approach.

Ascl2 is a transcription factor responsible for the differentiation of the trophoblast lineage in normal placenta [30]. Van der Flier et al. first identified Ascl2 as specifically expressed in the Lgr5 positive stem cells in the crypt base and that Ascl2 controls the fate of these cells, and conditional loss of expression resulted in the precise elimination of this cell population in mice, identifying an essential role for Ascl2 in intestinal stem cell maintenance, conversely, ectopic expression of Ascl2 throughout the mouse intestinal epithelia induced crypt hyperplasia and ectopic crypt formation suggesting that Ascl2 drives a neoplastic phenotype [11]. Additionally, in vitro data suggest that decreased expression of Ascl2 in HT-29 cells via Ascl2 interference results in arrest at the G2/M cell cycle checkpoint [10]. To our knowledge, our study is the only publication to date that demonstrates that the selective blockade of Ascl2 expression in HT-29 and LS174T cells results in tumor growth arrest both in vitro and in vivo, possibly through a miRNA-302b-related inhibition of colon cancer progenitor cells.

Ascl2 expression in CD133^+^ HT-29 cells was significantly higher than in CD133^−^ HT-29 cells. shRNA interference of Ascl2 expression in HT-29 cells decreases cellular proliferation, invasion, and migration ability in vitro and tumorigenic potential in vivo compared with control cells. Ascl2 blockade in HT-29 cells led to the significant reduction of CD133^+^ cells compared with control (26.2% verse 54.7%). Furthermore, expression levels of “stemness” associated genes, such as CD133, Lgr5, Oct4, Bmi1, Sox2, and C-myc were significantly decreased in shRNA-Ascl2/HT-29 and shRNA-Ascl2/LS174T cells compared with control (CD133 was not performed in shRNA-Ascl2/LS174T cells). Furthermore, Ascl2 selective blockade led to significant inhibition of tumorsphere formation in shRNA-Ascl2/HT-29 cells compared with shRNA-Ctr/HT-29 cells. Thus, our results demonstrate that Ascl2 participated in stemness maintenance of cancer progenitor cells and that Ascl2 interference led to HT-29 cells tumor growth arrest in vitro and in vivo through suppression of the stemness of colon cancer progenitor cells present in the repertoire of HT-29 cells. These results suggest that Ascl2 is a valuable candidate to target the cancer progenitor cells within colon cancers.

Several studies have demonstrated the significance of miRNAs in stem cell self-renewal and differentiation. Recently, miRNAs were shown to contribute to the regulation of stemness of human CSCs [26]–[28]. miR-200c provides a molecular link connecting normal stem cells with breast CSCs [31], whereas miR-451 inhibits tumor growth of glioblastoma stem cells [19]. These studies demonstrate that miRNAs have crucial regulatory functions in various CSCs. miRNAs generally affect downstream molecules by regulating the expression of target genes. Estimates suggest that ∼1–4% of genes in the human genome encode miRNAs, whereas a single miRNA can regulate as many as 200 mRNAs. The expression of miRNAs can be activated and repressed by transcription factors (TFs), which can serve as upstream regulators of miRNAs [32]. However, the study of miRNA regulation by TFs has been relatively limited. For example, p53 induces expression of miR-34a, which in turn suppresses of SIRT1 and thus increases p53 activity [33]. Therefore, TF-miRNA regulation is an important aspect in the study of miRNAs/TFs interactions and has been recently attracting the interest of researchers. Previously, the mechanism of participation of Ascl2 in stemness maintenance of colon CSCs through regulating miRNA was unknown. In this study, we performed miRNA arrays to examine the miRNA expression profiles of shRNA-Ctr/HT-29 cells and shRNA-Ascl2/HT-29 cells to determine potential players in the maintenance “stemness” by Ascl2. As a result, the microarray identified 84 differentially expressed miRNAs in shRNA-Ascl2/HT-29 cells compared with shRNA-Ctr/HT-29 cells. Interestingly, let-7b, miRNA-124, miRNA-125b were significantly up-regulated, whereas, miRNA-17, miRNA-20a, and miRNA-302b were significantly down-regulated. These miRNAs are involved in the stemness maintenance of stem cells [28], [34]–[37].

The let-7b, miR-124, miR-125b inhibitors and miR-17, miR-20a mimics did not affect the self-renewal of shRNA-Ascl2/HT-29 cells based on tumorsphere-formation assays, whereas miR-302b mimic restored the tumorsphere formation and cell ‘stemness’. Interestingly, Ascl2 levels, including mRNA and protein, were significantly increased when using miR-302b mimic in shRNA-Ascl2/HT-29 cells with significantly reduced Ascl2 expression due to Ascl2 interference. Based on this observation, we propose a positive feedback loop where Ascl2 inhibits expression of miR-302b, which increases Ascl2 levels. Presently we did not have the data whether Ascl2 influences miR-302b expression in a direct or non-direct effect, a further experiment is needed to explore how Ascl2 regulates miR-302b gene expression, most probably through transricptinal mechanism.

## Supporting Information

Table S1 **The primary antibodies used in the experiment.**
(DOC)

Table S2 **The primer sequences used in the real-time PCR experiment.**
(DOC)

Table S3 **The primer sequences used in the microRNA qPCR.**
(DOC)

Table S4 **2.0 fold upregulated miRNAs of shRNA-Ascl2/HT-29 cells versus shRNA-Ctr/HT-29 cells.**
(DOC)

Table S5 **2.0 fold downregulated miRNAs of shRNA-Ascl2/HT-29 cells versus shRNA-Ctr/HT-29 cells.**
(DOC)
