# Supplementary material for: Ascl2 Knockdown Results in Tumor Growth Arrest by miRNA-302b-Related Inhibition of Colon Cancer Progenitor Cells
Source: PLoS One. 2012 Feb 23;7(2):e32170. doi: 10.1371/journal.pone.0032170 (PMC3285660; doi:10.1371/journal.pone.0032170)
Supplement: Table S1 — The primary antibodies used in the experiment. (DOC) [file pone.0032170.s001.doc]

**Table S1 The primary antibodies** used in the experiment

| Primary antibody | Protein molecular weight | Companies purchased from | Dilution |
| --- | --- | --- | --- |
| Mouse monoclonal antibody to Ascl2 | 20 kDa | Millipore (MAB4417) | WB: 1:1000  IHC: 1:100 |
| Mouse monoclonal antibody to Bmi1 | 37 kDa | Abcam (ab14389) | WB: 1:1000 |
| Rabbit polyclonal antibody to Oct-4 | 45 kDa | [Cell Signaling Technology](http://www.cellsignal.com/) (#2750) | WB: 1:100 |
| Rabbit polyclonal antibody to CD133 | 110 kDa | Abcam (ab19898) | WB: 1:1000  IHC: 1:200 |
| Rabbit polyclonal antibody to Sox2 | 34 kDa | Chemicon (AB5603) | WB: 1:1000 |
| Mouse monoclonal antibody to Myc | 62 kDa | [Beyotime Institute of Biotechnology](http://www.google.com.hk/url?sa=t&source=web&cd=1&ved=0CCIQFjAA&url=http%3A%2F%2Fwww.beyotime.com%2F&ei=HdaFTYy9HovIuAO0hKC9CA&usg=AFQjCNE9yY2YEGhkVrRcrIEcjjp6S-ch5g) (AM926) | WB: 1:1000 |
| Mouse monoclonal antibody to Actin | 1. 42 kDa | [Beyotime Institute of Biotechnology](http://www.google.com.hk/url?sa=t&source=web&cd=1&ved=0CCIQFjAA&url=http%3A%2F%2Fwww.beyotime.com%2F&ei=HdaFTYy9HovIuAO0hKC9CA&usg=AFQjCNE9yY2YEGhkVrRcrIEcjjp6S-ch5g) (AA128) | WB: 1:1000 |
