# Supplementary material for: Ascl2 Knockdown Results in Tumor Growth Arrest by miRNA-302b-Related Inhibition of Colon Cancer Progenitor Cells
Source: PLoS One. 2012 Feb 23;7(2):e32170. doi: 10.1371/journal.pone.0032170 (PMC3285660; doi:10.1371/journal.pone.0032170)
Supplement: Table S2 — The primer sequences used in the real-time PCR experiment. (DOC) [file pone.0032170.s002.doc]

**Table S2 The primer sequences used** in the real-time PCR experiment

| Gene names | Forward | Reverse |
| --- | --- | --- |
| Ascl2  Lgr5  Sox2  Bmi1  CD133  Oct4  C-myc  β-catenin  β-actin | 5’-CGTGAAGCTGGTGAACTTGG-3’  5’-GGTGACAACAGCAGTATGGACGA-3’  5’-TGAGCGCCCTGCAGTACAA-3’  5’-GAGGGTACTTCATTGATGCCACAAC-3’  5’-AGTGGCATCGTGCAAACCTG-3’  5’-CTGGAGAAGGAGAAGCTGGA-3’  5’-GTCTCCACACATCAGCACAACT-3’  5’-AGTTGAGCACCTGTTTGCCTGA-3’  5’-TGGCACCCAGCACAATGAA-3’ | 5’-GGATGTACTCCACGGCTGAG-3’  5’-GAAGGTGAACACTGCACTGAATGAA-3’  5’-GCTGCGAGTAGGACATGCTGTAG-3’  5’-GCTGGTCTCCAGGTAACGAACAATA-3’  5’-CTCCGAATCCATTCGACGATAGTA-3’  5’-CAAATTGCTCGAGTTCTTTCTG-3’  5’-GTTCGCCTCTTGACATTCTCCT-3’  5’-ATGAGCAGCACTCGGACCTTC-3’  5’-CTAAGTCATAGTCCGCCTAGAAGCA-3’ |
