# Supplementary material for: Ascl2 Knockdown Results in Tumor Growth Arrest by miRNA-302b-Related Inhibition of Colon Cancer Progenitor Cells
Source: PLoS One. 2012 Feb 23;7(2):e32170. doi: 10.1371/journal.pone.0032170 (PMC3285660; doi:10.1371/journal.pone.0032170)
Supplement: Table S3 — The primer sequences used in the microRNA qPCR. (DOC) [file pone.0032170.s003.doc]

**Table S3 The primer sequences used in the microRNA qPCR**

| Gene names | Primer paires | Annealing temperatures (℃) | Product length (bps) |
| --- | --- | --- | --- |
| U6 | F: 5’-GCTTCGGCAGCACATATACTAAAAT-3’  R: 5’-CGCTTCACGAATTTGCGTGTCAT-3’ | 60 | 89 |
| hsa-miR-124 | F: 5'-GGTAAGGCACGCGGT-3’  R: 5'-CAGTGCGTGTCGTGGAGT-3' | 60 | 62 |
| hsa-miR-125b | F: 5’-GCTCCCTGAGACCCTAAC-3’  R: 5'-CAGTGCGTGTCGTGGAGT-3' | 60 | 66 |
| hsa-miR-302b | F: 5'-GGGTAAGTGCTTCCATGTTT-3'  R: 5'-CAGTGCGTGTCGTGGAGT-3' | 60 | 66 |
| hsa-miR-20a | F: 5'-GGGTAAAGTGCTTATAGTGC-3'  R: 5'-TGCGTGTCGTGGAGTC-3' | 60 | 63 |
| hsa-miR-17 | F: 5'-GGGCAAAGTGCTTACAGTG-3'  R: 5'-CAGTGCGTGTCGTGGAGT-3' | 60 | 66 |
| hsa-let-7b | F: 5'-GGGGTGAGGTAGTAGGTTG-3'  R: 5'-TGCGTGTCGTGGAGTC-3' | 60 | 63 |

F: forward primers, R: reverse primers.
