# Supplementary material for: Ascl2 Knockdown Results in Tumor Growth Arrest by miRNA-302b-Related Inhibition of Colon Cancer Progenitor Cells
Source: PLoS One. 2012 Feb 23;7(2):e32170. doi: 10.1371/journal.pone.0032170 (PMC3285660; doi:10.1371/journal.pone.0032170)
Supplement: Table S4 — 2.0 fold upregulated miRNAs of shRNA-Ascl2/HT-29 cells versus shRNA-Ctr/HT-29 cells. (DOC) [file pone.0032170.s004.doc]

**Table S4 2.0 fold upregulated miRNAs of shRNA-Ascl2/HT-29 cells versus shRNA-Ctr/HT-29 cells**

| ID | Name | FoldChange | ForeGround | | ForeGround-BackGround | | Normalized | |
| --- | --- | --- | --- | --- | --- | --- | --- | --- |
|  |  | shRNA-Ascl2/ shRNA-Ctr | shRNA-Ctr | shRNA-Ascl2 | shRNA-Ctr | shRNA-Ascl2 | shRNA-Ctr | shRNA-Ascl2 |
| 42769 | **Has-let-7b*** | 2.1058201 | 158.5 | 265 | 97.5 | 195 | 0.2449749 | 0.515873 |
| 30787 | **Has-miR-125b** | 3.2731769 | 113.5 | 280 | 69 | 214.5 | 0.1733668 | 0.5674603 |
| 42965 | Has-miR-424 | 2.2142079 | 83 | 140 | 34 | 71.5 | 0.0854271 | 0.1891534 |
| 146091 | Has-miR-1914 | 2.0367768 | 76.5 | 117.5 | 30.5 | 59 | 0.0766332 | 0.1560847 |
| 10943 | Has-miR-136 | 2.8007407 | 75 | 135.5 | 25 | 66.5 | 0.0628141 | 0.1759259 |
| 42773 | ebv-miR-BART17-3p | 2.4129189 | 94.5 | 171 | 48 | 110 | 0.120603 | 0.2910053 |
| 146165 | Has-miR-1973 | 3.0554543 | 682.5 | 1886 | 627 | 1819.5 | 1.5753769 | 4.8134921 |
| 148384 | Has-miR-3648 | 3.0222418 | 88 | 152.5 | 27 | 77.5 | 0.0678392 | 0.2050265 |
| 46259 | Has-miR-885-5p | 2.0612525 | 138 | 247 | 94.5 | 185 | 0.2374372 | 0.489418 |
| 147804 | Hsv1-miR-H17 | 2.1246221 | 73.5 | 117 | 28 | 56.5 | 0.0703518 | 0.1494709 |
| 147800 | Has-miR-2355-5p | 2.518726 | 71.5 | 122.5 | 25.5 | 61 | 0.0640704 | 0.1613757 |
| 148228 | Has-miR-3656 | 2.541507 | 89 | 169 | 43.5 | 105 | 0.1092965 | 0.2777778 |
| 147925 | Has-miR-3126-5p | 2.1706146 | 86 | 137 | 32.5 | 67 | 0.0816583 | 0.1772487 |
| 11184 | Has-miR-99b | 2.1254753 | 1628.5 | 3211.5 | 1553.5 | 3136 | 3.9032663 | 8.2962963 |
| 42654 | Has-miR-483-5p | 2.5219703 | 172 | 321.5 | 105 | 251.5 | 0.2638191 | 0.6653439 |
| 46567 | Has-miR-3176 | 2.8634017 | 123 | 184 | 41 | 111.5 | 0.1030151 | 0.2949735 |
| 28950 | Has-miR-455-3p | 3.0663696 | 357.5 | 975 | 313.5 | 913 | 0.7876884 | 2.4153439 |
| 14328 | **Has-miR-124** | 3.0777371 | 353.5 | 956 | 305.5 | 893 | 0.7675879 | 2.3624339 |
| 42823 | Has-miR-27b* | 2.9802708 | 76 | 148 | 29.5 | 83.5 | 0.0741206 | 0.2208995 |
| 145971 | Has-miR-611 | 2.1268783 | 70 | 110 | 25 | 50.5 | 0.0628141 | 0.1335979 |
| 147889 | Hsv1-miR-H14-3p | 5.3783784 | 173.5 | 631 | 111 | 567 | 0.2788945 | 1.5 |
| 148688 | Has-miR-765 | 2.0695129 | 98 | 123 | 29 | 57 | 0.0728643 | 0.1507937 |
| 148000 | Has-miR-3195 | 6.5201596 | 152 | 643 | 93.5 | 579 | 0.2349246 | 1.531746 |
| 146160 | Has-miR-133b | 2.0191099 | 135 | 220 | 85 | 163 | 0.2135678 | 0.4312169 |
| 17918 | Has-miR-222* | 2.5162766 | 86.5 | 134 | 29.5 | 70.5 | 0.0741206 | 0.1865079 |
| 42656 | kshv-miR-K12-10a | 2.090105 | 78 | 119.5 | 33.5 | 66.5 | 0.0841709 | 0.1759259 |

shRNA-Ascl2: shRNA-Ascl2/HT-29 cells; shRNA-Ctr: shRNA-Ctr/HT-29 cells; miRNAs marked as bold were selected for further experiments.
