# Supplementary material for: Ascl2 Knockdown Results in Tumor Growth Arrest by miRNA-302b-Related Inhibition of Colon Cancer Progenitor Cells
Source: PLoS One. 2012 Feb 23;7(2):e32170. doi: 10.1371/journal.pone.0032170 (PMC3285660; doi:10.1371/journal.pone.0032170)
Supplement: Table S5 — 2.0 fold downregulated miRNAs of shRNA-Ascl2/HT-29 cells versus shRNA-Ctr/HT-29 cells. (DOC) [file pone.0032170.s005.doc]

**Table S5 2.0 fold downregulated miRNAs of shRNA-Ascl2/HT-29 cells versus shRNA-Ctr/HT-29 cells**

| ID | Name | FoldChange | ForeGround | | ForeGround-BackGround | | Normalized | |
| --- | --- | --- | --- | --- | --- | --- | --- | --- |
|  |  | shRNA-Ascl2/ shRNA-Ctr | shRNA-Ctr | shRNA-Ascl2 | shRNA-Ctr | shRNA-Ascl2 | shRNA-Ctr | shRNA-Ascl2 |
| 42503 | hsa-miR-362-5p | 0.4774825 | 138.5 | 107.5 | 86 | 39 | 0.2160804 | 0.1031746 |
| 147837 | hsa-miR-3119 | 0.4084015 | 140 | 98.5 | 82.5 | 32 | 0.2072864 | 0.0846561 |
| 42667 | **hsa-miR-302b*** | 0.1660741 | 200 | 82 | 158.5 | 25 | 0.3982412 | 0.0661376 |
| 145734 | hsa-miR-33b* | 0.4725659 | 106 | 88 | 63.5 | 28.5 | 0.1595477 | 0.0753968 |
| 14285 | hsa-miR-487b | 0.3663785 | 362.5 | 176 | 307.5 | 107 | 0.7726131 | 0.2830688 |
| 146098 | hsv1-miR-H5-3p | 0.0930678 | 1284.5 | 183.5 | 1227.5 | 108.5 | 3.0841709 | 0.287037 |
| 147608 | hsa-miR-4307 | 0.3772928 | 114.5 | 94 | 60 | 21.5 | 0.1507538 | 0.0568783 |
| 17886 | hsa-miR-301b | 0.484855 | 969.5 | 490.5 | 917.5 | 422.5 | 2.3052764 | 1.1177249 |
| 46789 | hsa-miR-513b | 0.4607785 | 304 | 174 | 252.5 | 110.5 | 0.6344221 | 0.292328 |
| 42615 | hsa-miR-135b* | 0.2390041 | 127 | 80.5 | 81.5 | 18.5 | 0.2047739 | 0.0489418 |
| 14272 | hsa-miR-542-3p | 0.4076816 | 207 | 123 | 164 | 63.5 | 0.4120603 | 0.1679894 |
| 147793 | hsa-miR-4252 | 0.4825838 | 107.5 | 90 | 60 | 27.5 | 0.1507538 | 0.0727513 |
| 147554 | hsa-miR-4273 | 0.4401997 | 134.5 | 100 | 88.5 | 37 | 0.2223618 | 0.0978836 |
| 145742 | hsa-miR-935 | 0.3606653 | 233.5 | 146 | 181 | 62 | 0.4547739 | 0.1640212 |
| 147902 | hsa-miR-3193 | 0.4642753 | 122.5 | 106 | 63.5 | 28 | 0.1595477 | 0.0740741 |
| 10936 | hsa-miR-130b | 0.4987771 | 1888 | 942.5 | 1835.5 | 869.5 | 4.611809 | 2.3002646 |
| 17825 | hsa-miR-338-5p | 0.3912693 | 292.5 | 163 | 239.5 | 89 | 0.6017588 | 0.2354497 |
| 42839 | hsa-miR-135a | 0.2264323 | 206.5 | 100 | 139.5 | 30 | 0.3505025 | 0.0793651 |
| 145714 | hsa-miR-28-3p | 0.4736742 | 247.5 | 158 | 194.5 | 87.5 | 0.4886935 | 0.2314815 |
| 147649 | hsa-miR-500a/hsa-miR-500b | 0.4417804 | 199.5 | 128.5 | 143 | 60 | 0.3592965 | 0.1587302 |
| 148379 | hsa-miR-3654 | 0.4127115 | 1065 | 468 | 1009 | 395.5 | 2.5351759 | 1.0462963 |
| 42490 | hsa-miR-505* | 0.3026561 | 1006 | 341.5 | 948 | 272.5 | 2.3819095 | 0.7208995 |
| 13148 | hsa-miR-195 | 0.3479574 | 187.5 | 104 | 116.5 | 38.5 | 0.2927136 | 0.1018519 |
| 148221 | hsa-miR-3650 | 0.2030612 | 123.5 | 85.5 | 70 | 13.5 | 0.1758794 | 0.0357143 |
| 147688 | hsa-miR-548v | 0.3239723 | 111 | 86.5 | 52 | 16 | 0.1306533 | 0.042328 |
| 46440 | hsa-miR-1287 | 0.2094266 | 417 | 138.5 | 364.5 | 72.5 | 0.9158291 | 0.1917989 |
| 11092 | hsa-miR-378* | 0.2820295 | 108 | 76.5 | 56 | 15 | 0.1407035 | 0.0396825 |
| 42532 | hsa-miR-22* | 0.4456396 | 502 | 249.5 | 456 | 193 | 1.1457286 | 0.510582 |
| 147823 | hsa-miR-3146 | 0.0615497 | 687 | 97.5 | 641.5 | 37.5 | 1.611809 | 0.0992063 |
| 148643 | hsa-miR-642a | 0.1734205 | 126 | 70.5 | 85 | 14 | 0.2135678 | 0.037037 |
| 148620 | hsa-miR-454 | 0.4149179 | 636.5 | 290 | 590 | 232.5 | 1.4824121 | 0.6150794 |
| 11104 | hsa-miR-422a | 0.3495828 | 175 | 103 | 126.5 | 42 | 0.3178392 | 0.1111111 |
| 145845 | **hsa-miR-20a** | 0.463167 | 15463.5 | 6845 | 15414 | 6780.5 | 38.728643 | 17.937831 |
| 13143 | hsa-miR-301a | 0.3147404 | 5357.5 | 1644.5 | 5299 | 1584 | 13.31407 | 4.1904762 |
| 42739 | hsa-miR-339-5p | 0.4291257 | 2943 | 1235 | 2883 | 1175 | 7.2437186 | 3.1084656 |
| 42475 | hsa-miR-221* | 0.3906497 | 366.5 | 182 | 314 | 116.5 | 0.7889447 | 0.3082011 |
| 42592 | hsa-miR-338-3p | 0.4897787 | 6053.5 | 2850 | 6000 | 2791 | 15.075377 | 7.3835979 |
| 17570 | hsa-miR-589* | 0.1515552 | 110 | 73.5 | 66 | 9.5 | 0.1658291 | 0.0251323 |
| 46870 | hsa-miR-320d | 0.3719843 | 1729.5 | 655.5 | 1678.5 | 593 | 4.2173367 | 1.5687831 |
| 42466 | ebv-miR-BART18-3p | 0.065317 | 657 | 99.5 | 604.5 | 37.5 | 1.5188442 | 0.0992063 |
| 42902 | hsa-miR-185 | 0.4900141 | 2094.5 | 1007 | 2037 | 948 | 5.1180905 | 2.5079365 |
| 42783 | hsa-miR-197 | 0.4815851 | 996.5 | 492 | 944.5 | 432 | 2.3731156 | 1.1428571 |
| 42827 | hsa-miR-652 | 0.4595372 | 1058 | 500 | 1007 | 439.5 | 2.5301508 | 1.1626984 |
| 146112 | hsa-miR-30b | 0.4543559 | 10092 | 4394 | 10040 | 4332.5 | 25.226131 | 11.46164 |
| 145640 | hsa-miR-328 | 0.4296357 | 139 | 98 | 87 | 35.5 | 0.218593 | 0.0939153 |
| 145648 | hsa-miR-598 | 0.4615496 | 129 | 93 | 73 | 32 | 0.1834171 | 0.0846561 |
| 148624 | hsa-miR-942 | 0.3899667 | 117 | 87.5 | 67.5 | 25 | 0.169598 | 0.0661376 |
| 148668 | hsa-miR-378 | 0.3198369 | 2168.5 | 706 | 2125 | 645.5 | 5.339196 | 1.707672 |
| 148377 | hsa-miR-3653 | 0.3789648 | 4891 | 1803.5 | 4833 | 1739.5 | 12.143216 | 4.6018519 |
| 14307 | hsa-miRPlus-I382* | 0.3647335 | 119 | 77.5 | 76.5 | 26.5 | 0.1922111 | 0.0701058 |
| 11078 | hsa-miR-365 | 0.377903 | 2548.5 | 955 | 2502 | 898 | 6.2864322 | 2.3756614 |
| 46801 | hsa-miR-106a | 0.4709893 | 7055 | 3193 | 7009.5 | 3135.5 | 17.611809 | 8.2949735 |
| 46777 | **hsa-miR-17** | 0.4641108 | 7704.5 | 3431 | 7659 | 3376 | 19.243719 | 8.9312169 |
| 42477 | hsa-miR-324-5p | 0.413798 | 287 | 150 | 243 | 95.5 | 0.6105528 | 0.2526455 |
| 10987 | hsa-miR-193b | 0.4254945 | 2136.5 | 901.5 | 2091 | 845 | 5.2537688 | 2.2354497 |
| 145914 | hsa-miR-135b | 0.1125311 | 1317 | 188.5 | 1272.5 | 136 | 3.1972362 | 0.3597884 |
| 147755 | hsa-miR-378c | 0.2125258 | 857.5 | 217.5 | 812.5 | 164 | 2.0414573 | 0.4338624 |
| 148652 | hsa-miR-620 | 0.2178435 | 290.5 | 105.5 | 246.5 | 51 | 0.6193467 | 0.1349206 |

shRNA-Ascl2: shRNA-Ascl2/HT-29 cells; shRNA-Ctr: shRNA-Ctr/HT-29 cells; miRNAs marked as bold were selected for further experiments.
